# Neural and Behavioral Correlates of Sacred Values and Vulnerability to Violent Extremism

**DOI:** 10.3389/fpsyg.2018.02462

**Published:** 2018-12-21

**Authors:** Clara Pretus, Nafees Hamid, Hammad Sheikh, Jeremy Ginges, Adolf Tobeña, Richard Davis, Oscar Vilarroya, Scott Atran

**Affiliations:** ^1^Artis International, Scottsdale, AZ, United States; ^2^Departament de Psiquiatria i Medicina Legal, Universitat Autònoma de Barcelona, Cerdanyola del Vallès, Spain; ^3^Institut Hospital del Mar d’Investigacions Mèdiques (IMIM), Barcelona, Spain; ^4^Department of Security and Crime Science, University College London, London, United Kingdom; ^5^Department of Psychology, New School for Social Research, New York, NY, United States; ^6^School of Politics and Global Studies, Arizona State University, Tempe, AZ, United States; ^7^The Changing Character of War Centre, Pembroke College, University of Oxford, Oxford, England; ^8^Centre National de la Recherche Scientifique, Institut Jean Nicod – Ecole Normale Supérieure, Paris, France; ^9^Gerald Ford School of Public Policy and Institute for Social Research, University of Michigan, Ann Arbor, MI, United States

**Keywords:** sacred values, will to fight, social exclusion, Cyberball, neuroimaging, fMRI, radicalization, violent extremism

## Abstract

Violent extremism is often explicitly motivated by commitment to abstract ideals such as the nation or divine law—so-called “sacred” values that are relatively insensitive to material incentives and define our primary reference groups. Moreover, extreme pro-group behavior seems to intensify after social exclusion. This fMRI study explores underlying neural and behavioral relationships between sacred values, violent extremism, and social exclusion. Ethnographic fieldwork and psychological surveys were carried out among 535 young men from a European Muslim community in neighborhoods in and around Barcelona, Spain. Candidates for an fMRI experiment were selected from those who expressed willingness to engage in or facilitate, violence associated with jihadist causes; 38 of whom agreed to be scanned. In the scanner, participants were assessed for their willingness to fight and die for in-group sacred values before and after an experimental manipulation using Cyberball, a toss ball game known to yield strong feelings of social exclusion. Results indicate that neural activity associated with sacred value processing in a sample vulnerable to recruitment into violent extremism shows marked activity in the left inferior frontal gyrus, a region previously associated with sacred values and rule retrieval. Participants also behaviorally expressed greater willingness to fight and die for sacred versus nonsacred values, consistent with previous studies of combatants and noncombatants. The social exclusion manipulation specifically affected nonsacred values, increasing their similarities with sacred values in terms of heightened left inferior frontal activity and greater expressed willingness to fight and die. These findings suggest that sacralization of values interacts with willingness to engage in extreme behavior in populations vulnerable to radicalization. In addition, social exclusion may be a relevant factor motivating violent extremism and consolidation of sacred values. If so, counteracting social exclusion and sacralization of values should figure into policies to prevent radicalization.

## Introduction

One critical element affecting an individual’s willingness to engage in violent conflict between groups is the type of values that are at stake. People are more ready to engage in violence to defend so-called “sacred values,” which are immune or resistant to material tradeoffs and are associated with deontic (duty-bound) reasoning, than for mundane values that are associated with utilitarian cost-benefit calculations and consequentialist reasoning (Ginges et al., 2011). Sacred values—whether religious or secular (e.g., Holy Land, The Nation)—have been identified as crucial in making conflicts intractable (Ginges et al., 2007; Dehghani et al., 2010; Atran and Ginges, 2012). In addition, our research on radicalization (Sheikh et al., 2016) and with frontline combatants (Gómez et al., 2017) has shown that sacred values are a key component in the making of “devoted actors” (Atran et al., 2007; Atran, 2016), that is, those who are willing to make costly sacrifices, including giving their lives, for their cause. Sacred and nonsacred values have important distinctions in how they are processed and acted upon (Rappaport, 1971; Tetlock, 2003; Graham and Haidt, 2013). Sacred values tend to be highly stable, inspire costly sacrifices, and are difficult to socially influence (Sheikh et al., 2013).

It is also important to understand which factors may lead to a hardening of in-group values and resultant attitudes and actions. In particular, social exclusion has been shown to intensify in-group loyalty through extreme pro-group behavior in individuals who highly identify with their group (Gómez et al., 2011) and is related to pro-group (but not pro-self) unethical behavior (Thau et al., 2015) and aggressiveness more broadly (Leary et al., 2006 for a review). Thus, it is possible that social exclusion may encourage radicalization by increasing absolute willingness to defend even values that were not sacred at the outset of a conflict.

Research indicates that religious discrimination toward Muslim immigrants in Western Europe leads to economic and social exclusion (Adida et al., 2010), and when they feel marginalized (i.e., discriminated against and excluded), they increase support for radical groups (Lyons-Padilla et al., 2015). Combining our extensive ethnographic fieldwork with members of jihadist organizations—including reconstructing their life trajectories via interviews with their families, childhood friends, excoworkers, teachers, flatmates, and so forth—we have found that many begin their journeys toward political violence through a series of events that unmoor them from their immediate social surroundings (Atran, 2010; Hamid, 2017). In some cases, these can include breakdown in relations with family or other immediate members of their social network. The problem is especially acute for young adults from recent immigrant communities in Western Europe (Atran et al., 2017).

With these considerations in mind, the present studies were designed for a sample of young Muslim males in Europe vulnerable to recruitment into violent extremism. In view of previous studies and the gaps in our understanding, we sought to (1) test the generalizability of neural correlates for sacred (vs. nonsacred) values (Berns et al., 2012) in a population outside the Western mainstream; (2) determine if behavioral expressions of increased willingness to sacrifice for sacred (vs. nonsacred) values (Gómez et al., 2017) reliably extend to a population that supports values associated with militant jihadism; and (3) explore the effects of a social exclusion manipulation on behavioral and neural measures of sacred versus nonsacred values and willingness to fight and die. On the last point, the exploratory hypothesis was that social exclusion may lead to sacralization of values, thus increasing willingness to make costly sacrifices for them.

## Materials and Methods

### Participants

The ethnographic prestudy and field survey as well as the fMRI study were conducted in accordance with IRB Protocol #2014-0926, “The Neural Basis of Personal Beliefs: A Magnetic Resonance Imaging,” after approval by Artis Research IRB00007516, review by the U.S. Air Force Surgeon General’s Human and Animal Research Panel (SGHARP), and approval by the Comissió d’Ètica en l’Experimentació Animal i Humana (CEEAH), Universitat Autònoma de Barcelona, Ref. No. 2602, July 25, 2014. Oral informed consent was obtained from all participants in the behavioral study and again by all participants in the fMRI study. Oral over written informed consent was chosen both to ensure participants’ complete anonymity and to reduce any potential fears involved with signing a formal document that could be “potentially linked to authorities,” which is a prevalent worry in studies conducted in conflict settings (Moss et al., 2018).

Data protection was a priority, and complete anonymity was guaranteed along with explicit assurance that interviews or experiments involving answers to questionnaires could be terminated at will. The Artis IRB and the United States Air Force Surgeon General determined that signed and written signed informed consent procedures should be waved to protect confidentiality and security of subjects. Therefore, only consent was elicited, and no written or electronic records of names or other identifying information kept, ensuring that no outside agencies or authorities would be able to identify or track participants (Moss et al., 2018).

#### Ethnographic Prestudy and Survey

To select participants vulnerable to recruitment into violent extremism for the fMRI study and to develop ecologically valid and culturally relevant stimuli, we conducted ethnographic fieldwork from mid-2016 through mid-2017 with first- and second-generation young men from the Moroccan community in the province of Barcelona.

The Moroccan diaspora represents an immigrant community particularly prone to jihadist forms of radicalization both in Europe, in general (Rabasa and Benard, 2015), and in Spain, in particular (García-Calvo and Reinares, 2013). Spain hosts Europe’s second largest Moroccan diaspora community (after France) and its least integrated (Cemberero, 2009), whereas Catalonia hosts the largest and least integrated Moroccan community in Spain (Navarro, 2017). Barcelona, capital of Catalonia, was most recently the site of a mass killing in August 2017 by a group of young Moroccan men pledging support for the Islamic State. According to a recent Europol’s latest annual report on terrorism trends, Spain had the second highest number of jihadist terrorism-related arrests in Europe (second only to France) in 2016 and has consistently been ranked in previous annual reports as one of the EU’s main terrorism hubs over the last several years (EU Terrorism Situation and Trend Report (TE-SAT) 2017, 2017). The province of Barcelona is Spain’s primary hotspot for recruitment to violent extremist groups from a vulnerable population that is disproportionately (1) young, (2) Moroccan, (3) male, and (4) with criminal pasts (Reinares and García-Calvo, 2016). Neighborhoods that matched these demographics were actively probed for survey collection in specific Moroccan communities within the province of Barcelona.

We studied young men because they are significantly more susceptible (1) to commit severe violence than other age grades (Perkins, 1997; Ellis et al., 2009; Junger-Tas et al., 2012) or gender in general (Eagly and Steffen, 1986; Vugt et al., 2007) and (2) to recruitment into jihadi violence in particular (although nearly one-fourth to one-third of ISIS recruits in parts of Europe were female, the highest recorded for a jihadi group, Atran and Hamid, 2015). The comparative study of radicalization of women versus men is an important topic, given that women are less likely than men to commit violence against strangers or in groups (Kellermann and Mercy, 1992; Jacques and Taylor, 2008) yet more likely to play facilitating roles in social networks that link up militants and plotters that are not apparent to military or law enforcement (Atran and Hamid, 2016; Gaub and Lisiecka, 2016). However, such inquiry lies beyond the scope of this particular study.

#### Survey Procedure

After some months of participant observation in these selected neighborhoods, we approached each prospective participant (chosen as randomly as feasible while walking through these neighborhoods) to acquire informed consent that also ensured anonymity. We then proceeded to administer the survey, which lasted on an average of 45–60 min and allowed for justifications of responses and other reflections on their neighborhood and the world at large. Prospective participants were compensated with 20 Euros for taking the survey. Participants were told that they could keep the money whether or not they finished it. The overwhelming majority of them did complete the survey.

The total ethnographic sample consisted of male respondents, *n* = 535, average age mean = 23.47 years (range 18–42). All respondents were Sunni Muslim Moroccan men.

#### Participant Selection Measures

Participants who expressed willingness to personally engage in, or facilitate, defense of values advocated by jihadist groups were selected for the fMRI study (fMRI sample pool), that is, those who expressed willingness to defend these values by engaging in violent protest (*n* = 233), financially supporting a nonstate militant group (*n* = 160), joining a nonstate militant group (*n* = 132), or fighting and dying on their own (*n* = 95), and items that were part of a longer scale on costly sacrifices adapted from field surveys in conflict zones (Gómez et al., 2017). Values advocated by violent jihadist groups included: “strict sharia should be applied to all Muslim lands,” “all current Muslim countries should be replaced by a single borderless Caliphate,” “armed jihad should be waged against the enemies of Muslims,” and the like. These values were generated from fieldwork with this community but also from previous survey research on violent radicalization (Sheikh et al., 2016; Gómez et al., 2017). This yielded a selection pool of 267 participants deemed vulnerable to recruitment into violent extremism.

#### Measures of Vulnerability to Violent Extremism

Compared with the rest of participants (others, *n* = 268), the participants in the fMRI pool (*n* = 267) scored higher on all measures of vulnerability to recruitment into violent extremism (see Table 1). These measures included (1) a modified inventory on general radicalization (support for violence as a political tactic) based on a prior longitudinal study on violent extremist attitudes among Swiss adolescents (Nivette et al., 2017); (2) a scale on personal grievances and previously used on imprisoned Islamist militants in the Philippines, and Tamil Tigers in Sri Lanka (Webber et al., 2018); (3) a scale on collective narcissism which has been shown to shape in-group authoritarian identity and support for military aggression against outgroups (de Zavala et al., 2009); (4) a self-report delinquency inventory adapted from Elliott et al. (1985), based on the disproportionate number of Muslim European delinquents who join jihadist terrorist groups (Basra and Neumann, 2016); and (5) a series of items assessing endorsement of militant jihadism (“The fighting of the Taliban, Al Qaida, ISIS is justified,” “The means of jihadist groups are justified,” “Attacks against Western nations by jihadist groups are justified,” “Attacks against Muslim nations by jihadist groups are justified,” “Attacks against civilians by jihadist groups are justified,” “Spreading Islam using force is every part of the world is an act of justifiable jihad,” and “A Caliphate must be resurrected even by force”) that we combined into a reliable composite score, “Endorsement of Militant Jihadism,” mean (SD) = 2.13(1.21), and Cronbach’s *α* = 0.83. Participants in the fMRI pool were also more likely (9%) to be “fused” with Mujahidin (Warriors of Islam—the name that jihadists give to themselves) than those not selected for the pool (3%), *χ* ^2^ (*n* = 525) = 8.00, *p* = 0.004, in line with previous literature linking identity fusion with a group (Swann et al., 2012), and willingness to commit extreme actions (Swann et al., 2009). The Cronbach’s α reliability scores of each scale are presented in Table 1.

**Table 1:** Behavioral comparison between the sample selected for the fMRI session (“fMRI pool”) and the rest of the field survey respondents (“Others”).

Scale	fMRI pool (*n* = 267) Mean (SD)	Others (*n* = 268) Mean (SD)	*t* statistic (*df* = 533)	*p-value*	Cohen’s *d*
General radicalization (range: 1–7) *α* = 0.84	3.74 (1.57)	2.20 (1.30)	12.42	<0.001	0.95
Personal grievance (range: 1–7) *α* = 0.75	2.56 (0.97)	2.28 (0.84)	3.61	<0.001	0.31
Collective narcissism (range: 1–7) *α* = 0.85	5.18 (1.25)	4.54 (1.51)	5.37	<0.001	0.45
Delinquency inventory (range: 1–12) *α* = 0.92	4.37 (3.59)	2.90 (3.36)	3.68^*^	<0.001	0.41
Endorsement of militant jihadism (range: 1–7) *α* = 0.83	2.56 (1.28)	1.70 (0.97)	8.78	<0.001	0.71

* Degrees of freedom: 302 (231 participants did not complete the delinquency inventory).

#### fMRI Participants

Of the 267 participants who met our selection criteria, 38 participants agreed to take part in our fMRI study. The scanned participants were statistically indistinguishable from the selection pool on most measures after the Bonferroni correction, except one, that is, they had higher scores on Endorsement of Militant Jihadism *t*(265) = 4.24, *p* < 0.001, mean (SD)_pool_ = 2.43 (1.13), mean(SD)_fMRI_ = 3.35 (1.26), and Cohen’s *d* = 0.72. Thus, if anything, they were more prone to violence and more aggrieved than the selection pool.

The ages of the 38 selected participants ranged between 18 and 27 years and was, on average, 19.37 years (SD = 2.31). The average score of the “Endorsement of Militant Jihad” composite was 3.35 (SD = 1.32) out of 7 points. Baseline aggression was 2.23 (SD = 0.60) out of 5 points at average; the sensitivity to rejection score was 6.82 (SD = 2.68) out of 36 points; the delinquency inventory score was 4.5 out of 12 possible crimes; all participants’ IQ scores fell within the normal range, as estimated by the WAIS-III Block design subscale (Wechsler, 1997). The excluded and control groups did not differ on any of these measures. Participants were screened for psychiatric disorders including substance abuse by means of the MINI interview (Sheehan et al., 1998).

### Experimental Procedure and Measures

**Figure 1:**
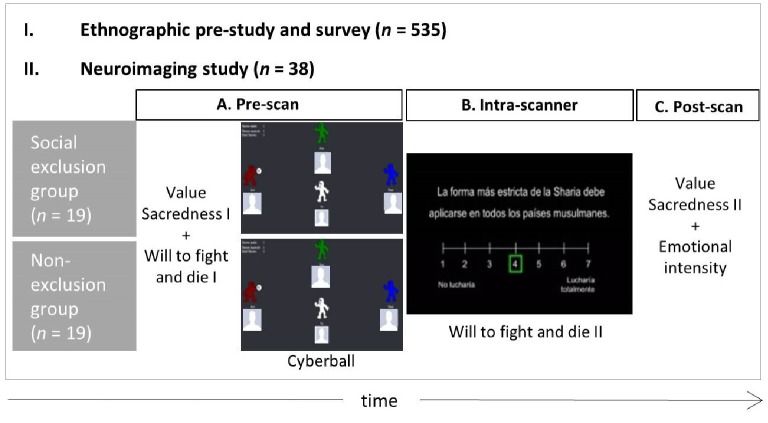
Scheme of the testing timeline consisting of (I) the ethnographic prestudy and survey and (II) the neuroimaging study including **(A)** a prescan behavioral testing session (around 45 min) including sacred value selection by rejection of material tradeoff, the premanipulation will to fight and die measure for sacred and nonsacred values, and the Cyberball experimental manipulation in either the social exclusion or the non-exclusion condition (between-subjects factor), **(B)** an intrascanner session (around 20 min) including a postmanipulation will to fight and die measure for sacred and nonsacred values, and **(C)** a postscan behavioral session (around 45 min) including sacredness reassessment of nonsacred values by rejection of material trade-off and emotional intensity scores at the thought of defending each value.

The experimental session consisted of three stages. In the prescan behavioral testing stage (Figure 1A), participants were assessed for value sacredness and for willingness to fight and die for values (premanipulation measure). Next, they completed a social exclusion manipulation in either one of two experimental conditions, social exclusion or control (i.e., social inclusion) by means of the online toss-ball game Cyberball (Williams and Jarvis, 2006). In the last stage, participants were instructed to convey their willingness to fight and die for the same values while remaining in the fMRI scanner (postmanipulation measure).

In line with previous studies, value sacredness was evaluated by rejection of a material trade-off presented in case-based scenarios (Fiske and Tetlock, 1997; Baron and Leshner, 2000; Ginges et al., 2007; Berns et al., 2012; Sheikh et al., 2013), that is, a value was considered sacred if participants were not willing to give up the value in exchange for instrumental benefits such as better economic conditions for their own community. Willingness to fight and die scores were also obtained for each value at that stage (premanipulation measure). The list of candidate sacred values was previously developed based on the ethnographic fieldwork and included religious, cultural, and political issues (e.g., Palestinian right of return, Western military forces being expelled from all Muslim lands, strict sharia as the rule of law in all Muslim countries, armed jihad being waged against enemies of Muslims, forbidding of caricatures of Prophet Mohammed, and veiling of women in public). The individualized battery of 6 sacred and 6 nonsacred values was then introduced in the intrascanner paradigm, together with 7–8 grammatical variations of each value. This produced 50 different items per condition (sacred and nonsacred).

Before commencing the intrascanner paradigm, participants completed the social exclusion manipulation Cyberball (Williams and Jarvis, 2006). Cyberball is an intervention known to yield strong effects, with Cohen’s *d* coefficients between 1 and 2. For instance, Williams and Jarvis (2006) report *p* values below *p* < 0.05 with as few as three participants per condition. The game was programmed to include three other players with a demographic profile similar to the study participants (young males), except with Spanish sounding names (“Dani,” “Jose,” and “Javi”) and typical Spanish faces represented by a profile picture (concealed in Figure 1A, owing to privacy restraints). Nineteen participants played the game in the social exclusion condition, where they received the ball 2 times out of 30 total throws (standard exclusion set up). The other 19 participants completed the social inclusion (or nonexclusion) condition, where they received the ball 1/3 of the time. The instructions of the game induced participants to believe that they were going to play against three real online players. The total duration of the game was around 2 min, slightly varying with the participant’s performance. This was followed by a standard Cyberball manipulation check questionnaire, including questions on the four “fundamental needs”: belonging, control, self-esteem, and meaningful existence, together with positive and negative mood.

The second stage, immediately after the social exclusion manipulation, involved completion of an intrascanner paradigm (Figure 1B). The intrascanner paradigm included one hundred 7-second randomized trials split into two runs, each lasting 8.5 min. For every trial, participants had to convey their willingness to fight and die to defend one of their sacred or nonsacred values appearing on screen in one of its grammatical forms (postmanipulation measure). The response scale ranged from 1 (“Not willing at all”) to 7 (“Extremely willing”). Participants introduced their responses by pressing two buttons (either left or right), shifting the cursor toward the desired response starting from the middle point (4 points). To distinguish missing values from “4” responses, participants were instructed to always move the cursor to convey a response. Before entering the fMRI scan, participants were trained to complete the task on a laptop using a collection of mock values, including issues not specific to the Muslim community (e.g., “Elderly people should be respected,” “Children should be protected from any harm”).

In the third stage, participants completed one last battery of questionnaires and behavioral tests (Figure 1C), including a postmanipulation sacredness reassessment of nonsacred values and an emotional intensity assessment (among other confounding factors). The sacredness reassessment of nonsacred values aimed to evaluate whether or not the sacred versus nonsacred value neural differences were related to actual value sacralization. For that purpose, the same case-based material trade-off scenarios used in the prescan stage were readministered only for nonsacred values. In addition, a series of potential confounds were tested for neural differences between sacred and nonsacred values, including familiarity (“I am quite familiar with this issue”), salience (“How frequently do ideas, thoughts, impulses, images related to this issue occur?”), attitude strength (“How certain are you about your position on this issue?”), and emotional intensity (“How much of each emotion do you experience at the thought of defending this idea?” including anger, joy, fear, and sadness). Familiarity, salience, attitude strength, and emotional intensity ratings were not available for one participant.

### Analysis and Results

#### Behavioral Analysis

The main behavioral analysis involved a repeated measure ANOVA on willingness to fight and die ratings with value sacredness (sacred vs. nonsacred) and pre/postmanipulation measures as within-subjects factors and group membership (social exclusion vs. social inclusion) as between-subjects factor. This analysis was aimed to capture any differences in willingness to fight and die stemming from the interaction between value sacredness and social exclusion.

Several secondary analyses were run to test whether social exclusion also affected emotional intensity ratings or value sacralization, and, if so, whether neural changes associated with social exclusion were correlated with changes in any of these factors. Thus, to test the interaction between exclusion and value sacredness on emotional intensity, a further repeated measure ANOVA was conducted on emotional intensity scores with only one within-subjects factor (sacred vs. nonsacred values) and one between-subjects factor (group membership). In addition, the percentage of nonsacred values that qualified as sacred in the postmanipulation reassessment was compared between groups. Postmanipulation sacred versus nonsacred value differences in willingness to fight and die, emotional intensity, and nonsacred value “sacralization” were then correlated with neural differences between sacred and nonsacred values.

#### Behavioral Results

**Figure 2:**
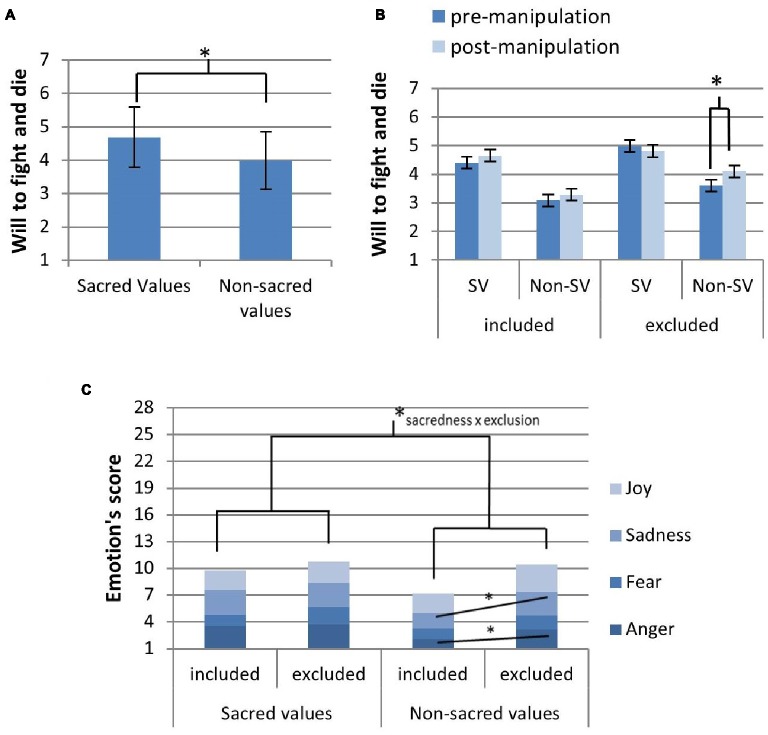
Behavioral analysis results including: **(A)** higher premanipulation will to fight and die for sacred versus nonsacred values (*t*(37) = 10.831, *p* < 0.001, and Cohen’s *d* = 0.80), **(B)** an increase in will to fight and die for nonsacred versus sacred values in excluded participants compared to nonexcluded participants after the experimental manipulation (Wilks’ *λ* = 0.852, *F*(1,35) = 6.077, *p* = 0.019, and *η* ^2^_*p*_ = 0.148), and **(C)** higher emotional intensity ratings (out of 28 possible points) at the thought of defending sacred versus nonsacred values (*t*(36) = 2.59, *p* = 0.014, and Cohen’s *d* = 0.40) and higher emotional intensity ratings for nonsacred values versus sacred values in the social exclusion versus inclusion condition (Wilks’ *λ* = 0.848, *F*(1,35) = 6.26, *p* = 0.017, *η* ^2^_*p*_ = 0.152), driven by sadness (Wilks’ *λ* = 0.834, *F*(1,35) = 6.99, *p* = 0.012, and *η* ^2^_*p*_ = 0.166) and anger (Wilks’ *λ* = 0.888, *F*(1,35) = 4.43, *p* = 0.043, and *η* ^2^_*p*_ = 0.112).

As anticipated, prior to the manipulation, participants conveyed higher willingness to fight and die for sacred values compared to nonsacred values (sacred values: mean (SD) = 4.69(0.91), nonsacred values: mean (SD) = 3.33(0.85), *t*(37) = 10.831, *p* < 0.001, Cohen’s *d* = 0.80, Figure 2A). Sacred versus nonsacred values also received higher ratings in familiarity (sacred values: mean (SD) = 4.87 (0.92); nonsacred values: mean (SD) = 3.99 (0.98), *t*(36) = 6.76, *p* < 0.001, and Cohen’s *d* = 0.93), salience (sacred values: mean (SD) = 2.44 (0.76); nonsacred values: mean (SD) = 2.22 (0.74), *t*(36) = 2.14, *p* = 0.039, and Cohen’s *d* = 0.31), attitude strength (sacred values: mean (SD) = 5.46 (1.10); nonsacred values: mean (SD) = 4.73 (1.21), *t*(36) = 4.94, *p* < 0.001, and Cohen’s *d* = 0.64), and emotional intensity ratings (sacred values: mean (SD) = 2.54 (0.74); nonsacred values: mean (SD) = 2.24 (0.80), *t*(36) = 2.59, *p* = 0.014, and Cohen’s *d* = 0.40). Only differences in familiarity and attitude strength survived the Bonferroni correction.

Standard manipulation checks for social exclusion were positive. During the manipulation, participants going through the social exclusion versus control condition reported decreased feelings of belongingness (excluded: mean (SD) = 9.10 (3.77); included: mean (SD) = 13.26 (4.31), *t*(36) = 3.167, and *p* = 0.003), being in control (excluded: mean (SD) = 7.79 (4.39); included: mean (SD) = 13.52 (4.65), *t*(36) = 3.911, and *p* < 0.001), self-esteem (excluded: mean (SD) = 8.95 (4.93); included: mean (SD) = 14.53 (4.50), *t*(36) = 3.644, and *p* = 0.001), and meaningful existence (excluded: mean (SD) = 8.63 (4.03); included: mean (SD) = 12.94 (3.83), *t*(35) = 3.334, and *p* = 0.002) with large effect sizes (Cohen’s *d* between 1.03 and 1.23). Despite the effects of social exclusion on emotional intensity ratings, exclusion did not affect participants’ positive and negative mood reports.

The social exclusion manipulation had a significant effect on willingness to fight and die and emotional intensity scores (Figures 2B,C). The interaction between value sacredness, pre and postmeasures, and the social exclusion condition was significant (Figure 2B). Here, socially excluded participants showed a significant increase in willingness to fight and die for nonsacred versus sacred values compared to nonexcluded participants in post versus premanipulation measures (Wilks’ *λ* = 0.852, *F*(1,35) = 6.077, *p* = 0.019, and *η* ^2^_*p*_ = 0.148). That is, excluded participants conveyed higher willingness to fight and die for nonsacred values after the social exclusion manipulation, whereas sacred values in excluded participants, and both sacred and nonsacred values among controls, did not show similar changes. These results did not stem from ceiling effects in the sacred value condition inasmuch as sacred value ratings still had, on average, more than a 2-point margin to rise (from the average rating 4.73 up to the maximal rating 7).

The interaction between value sacredness and social exclusion had a significant effect on emotional intensity associated with defending values (Wilks’ *λ* = 0.848, *F*(1,35) = 6.26, *p* = 0.017, and *η* ^2^_*p*_ = 0.152), which was driven by sadness (Wilks’ *λ* = 0.834, *F*(1,35) = 6.99, *p* = 0.012, and *η* ^2^_*p*_ = 0.166) and anger (Wilks’ *λ* = 0.888, *F*(1,35) = 4.43, *p* = 0.043, and *η* ^2^_*p*_ = 0.112). Of the 6 nonsacred values per participant, the number of nonsacred values fulfilling sacred value criteria after the experimental manipulation did not differ between groups (exclusion: mean(SD) = 1.54(1.34); inclusion: mean(SD) = 1.25(1.55), *t*(25) = −0.535, *p* = 0.597, and Cohen’s *d* = 0.21). Postmanipulation sacredness reassessment measures were only available for 27 participants.

#### Neuroimaging Data Acquisition and Analysis

Images were acquired in a Philips 3T scanner. T1-weighted images were obtained using a FSPGR sequence (TR: 9.9 ms, TE: 4.6 ms, FA: 8, matrix size: 240 × 240, 180 slices, and slice thickness: 1.00 mm). An EPI-T2* sequence allowed obtaining the functional volumes (TR: 1600 ms, TE: 35 ms, FA: 70, matrix size: 76 × 76, 46 slices, and slice thickness: 3.1 mm).

The neuroimaging analysis was conducted by means of SPM12 (Wellcome Trust Centre for Neuroimaging, UCL, London, United Kingdom). Functional images were realigned and coregistered to the structural images, which were then segmented into white matter, gray matter, and cerebrospinal fluid. The forward deformation fields generated during the segmentation of the structural images were used to normalize the functional images. Finally, images were smoothed using an 8-mm FWHM kernel.

Two different GLMs are presented. The main GLM aimed to capture effects on neural activity of value sacredness and willingness to fight and die. It included two regressors accounting for neural activity related to sacred and nonsacred values, respectively, each modeled by a parametric regressor that included willingness to fight and die ratings. A button press regressor and six movement regressors were also included in the model.

A second GLM was conducted to account for confounding factors potentially affecting the sacred versus nonsacred contrast. Aside from the sacred and nonsacred value regressors, four parametric regressors included as modulators of each sacred and nonsacred value condition, representing familiarity, salience, attitude strength, and emotional intensity scores. The button press and the six movement regressors were also added.

Individual sacred versus nonsacred values, as well as willingness to fight and die contrast images, were tested at a group level. To capture social exclusion effects on the sacred versus nonsacred value contrast, a two-sample *t* test was conducted on individual sacred versus nonsacred value contrast images with the Cyberball condition (social exclusion vs. inclusion) as between-subjects factor. This procedure was repeated using contrast images obtained from the second GLM to control for the effect of confounding factors on the sacred versus nonsacred value contrast.

For each contrast, a whole-brain analysis was conducted using a threshold of *p* < 0.05 corrected for multiple comparisons by means of the family-wise error rate (FWE) at a cluster level, with a peak-level threshold of *p* < 0.001.

To specifically test for brain activity differences associated with social reasoning during moral judgment across the experimental conditions (sacred vs. nonsacred values × social exclusion vs. inclusion), we reanalyzed the data with a temporoparietal junction (TPJ) mask extracted from Neurosynth. The right TPJ has been consistently associated with inference of other’s intentions during moral judgment, especially while conveying one’s own moral responses versus evaluating other’s moral responses (see Sevinc and Spreng, 2014 and Garrigan et al., 2016 for a meta-analysis). Temporal inhibition of the right TPJ using transcranial magnetic stimulation appears to increase acceptability of intentionally harming others (Young et al., 2010).

#### Neuroimaging Results

**Figure 3:**
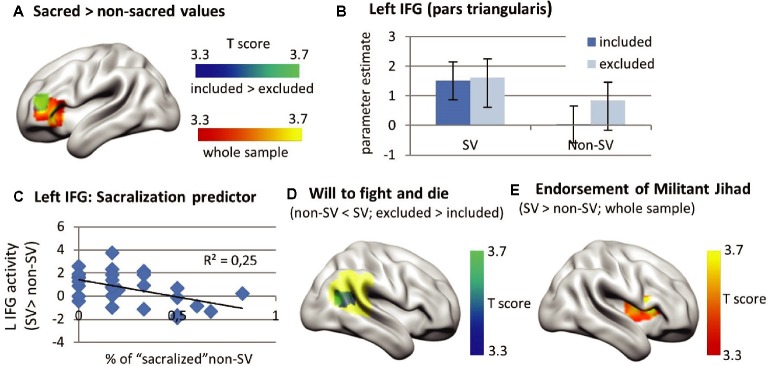
Neuroimaging results including **(A)** higher activity in the left inferior frontal gyrus (IFG, pars triangularis) in the sacred compared to the nonsacred condition in the whole sample (results in red-yellow) and higher between-condition difference in left IFG activity in the included compared to the excluded group (small volume correction with left IFG cluster obtained in the whole sample analysis), **(B)** similar left IFG activity estimates in the sacred value versus baseline contrast in both groups, but higher left IFG activity estimates in the nonsacred value versus baseline condition in the exclusion versus inclusion group, explaining between-group left IFG activity differences shown in **(A),** **(C)** a negative correlation between left IFG activity differences between sacred versus nonsacred values and percentage of nonsacred values that became sacred in the postmanipulation sacredness reassessment ( *r* = −0.496, *p* = 0.007, and *N* = 28), even after controlling for social exclusion ( *r* = −0.485 and *p* = 0.010), **(D)** significantly lower right temporoparietal activity in response to willingness to fight and die for nonsacred versus sacred values in excluded compared to included participants after small volume correction by means of a right TPJ mask extracted from Neurosynth (*T* = 4.34, *p* = 0.044 FWEc, single voxel *p* < 0.001, and mask shown in yellow), and **(E)** higher activity in the right insula (*T* = 5.30, *p* < 0.001 FWEc, and single voxel *p* < 0.001), left insula (*T* = 4.72, *p* = 0.005 FWEc, and single voxel *p* < 0.001), and right postcentral gyrus (*T* = 4.96, *p* = 0.004 FWEc, and single voxel *p* < 0.001) during the sacred versus nonsacred value condition predicted by the “Endorsement of Militant Jihad” score in the whole sample.

Neuroimaging results are shown in Table 2 and in Figure 3. The sacred versus nonsacred value contrast yielded significantly higher activity in the left inferior frontal gyrus (IFG, pars triangularis) for the sacred versus nonsacred condition in the whole fMRI sample (*T* = 4.80, *p* = 0.019 FWEc, single voxel *p* < 0.001, Figure 3A in red-yellow).

**Table 2:** Results of the neural analysis including the within-subject sacred and nonsacred value conditions each modelled by willingness to fight and die scores as a parametric regressor and social exclusion versus nonexclusion as a between-subjects factor.

*N* = 38	Region label (aal)	MNI coordinates			K	*T* max	*p-value*
		*x*	*y*	*z*			
**A. Sacred > nonsacred values**							
Whole sample	L inferior frontal gyrus (pars triangularis)	−46	26	−2	401	4.80	0.019
	R lingual	10	−68	−6	941	6.57	<0.001
Included > excluded	L inferior frontal gyrus (pars triangularis )	−38	42	12	23	4.91	0.016^*^
Excluded > included	–						
**B. Will to fight and die for nonsacred < sacred values (right TPJ mask)**							
Excluded > Included	R temporoparietal junction	44	−62	18	28	4.34	0.044
**C. Endorsement of Militant Jihad score predicting activity in the Sacred values > nonsacred values**							
Whole sample	R insula	30	16	12	879	5.30	<0.001
	R postcentral	48	−6	44	547	4.96	0.004
	L Heschl/L insula	−30	−30	8	514	4.72	0.005

* Small volume correction using left inferior frontal gyrus mask extracted from the whole sample sacred versus nonsacred value contrast.

When comparing the socially excluded group versus the control group, excluded participants exhibited lower differences in left IFG activity for the sacred versus nonsacred conditions, compared to controls (small volume correction with left IFG mask extracted from the whole sample analysis, *T* = 4.91, *p* = 0.016 FWEc, single voxel *p* < 0.001, see Figure 3A in blue-green). The results remained significant after accounting for variability associated with familiarity, salience, attitude strength, and emotional intensity of each value in the confounding factor analysis (second GLM). That analysis included the four scores as first-level parametric regressors in the sacred and nonsacred value conditions (see Supplementary Information, Table S1). The confounding factor analysis could only be done with 28 participants, owing to the lack of variability in any one of the four scales in nine participants, and missing data in one participant.

By extracting parameter estimates, we further explored between-group differences in left IFG activity. This revealed similarly high levels of left IFG activity in the sacred value versus baseline contrast in both groups, but higher left IFG activity in excluded versus control participants in the nonsacred versus baseline contrast (Figure 3B). The smaller the difference in left IFG activity between sacred and nonsacred values, the more that nonsacred values were reported as sacred in the postmanipulation sacredness reassessment (*r* = −0.496, *p* = 0.007, after exclusion of one outlier with left IFG activity differences 2 SD below average, Figure 3C). The sacred versus nonsacred value difference in left IFG activity was not correlated with either differences in willingness to fight and die or differences in emotional intensity.

Excluded compared to included participants exhibited significantly lower activity in the right temporoparietal junction (R TPJ) in response to willingness to fight and die for nonsacred values (vs. sacred values) after small volume correction using a temporoparietal junction mask from Neurosynth (Yarkoni et al., 2011) (see Table 2B and Figure 3D).

Finally, the group-level regression analysis revealed a significant correlation between the “Endorsement of Militant Jihad” score and activity in the right insula (*T* = 5.30, *p* < 0.001 FWEc, and single voxel *p* < 0.001), left insula (*T* = 4.72, *p* = 0.005 FWEc, single voxel *p* < 0.001) and right postcentral gyrus (*T* = 4.96, *p* = 0.004 FWEc, and single voxel *p* < 0.001) during the sacred versus nonsacred value contrast in the whole sample (see Table 2C and Figure 3E). Parameter estimate extraction revealed that the right insula activity predicted by the “Endorsement of Militant Jihad” score correlated with higher sacred versus nonsacred value differences in left inferior frontal gyrus (*r* = 0.459 and *p* = 0.004).

Complementary neural results for willingness to fight and die controlling for the effect of value sacredness were also computed (see Supplementary Material, Table S2, and Figure S1).

## Discussion

The present work offers an interdisciplinary perspective on vulnerability to violent extremism, combining ethnographic fieldwork, social psychology, and neuroimaging techniques. The field prestudy allowed for the selection of a real-world sample of a Muslim European community with traits of vulnerability to radicalization. The neuroimaging study enabled identification of the neural correlates of willingness to fight and die for sacred values after social exclusion compared to a nonexcluded control group.

We found that processing sacred versus nonsacred values was associated with activity in the left inferior frontal gyrus, an area previously related with sacred values and rule-bound thinking (Berns et al., 2012). After the social exclusion manipulation, nonsacred values showed higher levels of left inferior frontal gyrus activity that were more similar to sacred values compared to the nonexcluded condition. In terms of left IFG activity, the more similar non-sacred values became to sacred values, the more non-sacred values matched sacred value criteria after the experimental manipulation. Behaviorally, we found that simulating social exclusion increased willingness to fight and die for values that were not initially sacred, whereas sacred value ratings remained consistently high. We also found that, after exclusion, nonsacred values showed an increase in emotional intensity that matched sacred values. Together, these results suggest that nonsacred values become more similar to sacred values in terms of neural activity and willingness to fight and die when participants experience social exclusion.

At a neural level, sacred (vs. nonsacred) values in this young vulnerable sample appear to rely on left inferior frontal gyrus recruitment. Activity in this area has also been associated with sacred values in a sample “representative of the US typical population” (Berns et al., 2012). The left inferior frontal gyrus (pars triangularis) is a part of Bordmann’s area 45 and Broca’s area and has been typically associated with semantic rule retrieval and semantic working memory (Badre et al., 2005; Souza et al., 2009). Berns et al. (2012) argued that its role during sacred value processing could be that of “retrieval and processing of deontic rules” in opposition to utilitarian cost-benefit reasoning. In the present study, the left inferior frontal gyrus could be responding to deontic rule retrieval during sacred versus nonsacred value processing in our sample of young members of a European Muslim community. Moreover, our study rules out confounding factors such as familiarity and salience of the value, as well as attitude strength and emotional intensity at the thought of defending the value (scores which were all higher for sacred vs. nonsacred values).

With respect to the social exclusion manipulation, neural differences between the sacred and nonsacred value condition in the left IFG were lower in excluded versus control participants, that is, the left IFG responded more similarly to sacred and nonsacred values in the excluded (vs. control) group. In addition, the smaller the difference in left inferior frontal activity between sacred and nonsacred values, the more the nonsacred values fulfilled the sacred value criteria in the postmanipulation sacredness reassessment. Social exclusion (vs. inclusion) also effectively increased expressed willingness to fight and die ratings for nonsacred (vs. sacred) values, pulling willingness to fight and die scores closer to those of their sacred counterparts. Although the number of nonsacred values fulfilling sacred value criteria after exclusion versus inclusion did not reach statistical significance, both neural and behavioral findings suggest that social exclusion had a partial “sacralization effect” on otherwise nonsacred in-group values in terms of their neural signature and willingness to fight and die to defend them.

Finally, willingness to fight and die for nonsacred values (vs. sacred values) exhibited a decrease in temporoparietal junction activity in excluded participants. The right TPJ has been consistently associated with inference of other’s intentions while conveying one’s own moral responses versus evaluating those of others (see meta-analyses by Sevinc and Spreng, 2014; Garrigan et al., 2016). Thus, the fact that the right TPJ exhibited lower activity in response to willingness to fight and die for nonsacred values suggests a diminished role of deliberative social reasoning in moral judgment after social exclusion. Nevertheless, more research would be needed to clarify whether such TPJ decrease is (1) behaviorally related to decreased inference of other’s intentions while conveying willingness to fight and die after social exclusion and (2) whether it is related to increased acceptability of intentionally harming others (Young et al., 2010).

Our results are consistent with Kruglanski et al. (2013) Quest for Significance Theory of Radicalization, which argues that violent extremism in defense of the in-group is driven by the motivation to restore one’s personal significance or self-worth, often following episodes of significance loss or humiliation. In this situation, Kruglanski et al. (2014) found that “the individual comes to share in the violence-justifying ideology and proceeds to implement it as a means of significance gain,” including adoption of sacred values that the individual perceives to increase self-worth. According to Bélanger (2015), when the quest for significance is activated—“a phenomenon exacerbated by a threat to identity”—individuals seek the means to satiate this quest, including association with groups and values that enhance self-image and sentiments of importance and power. Interviews with would-be suicide bombers (as opposed to leaders and organizers of suicide attacks) also indicate “lower level of ego strength” that makes them more amenable to groups, leaders, and esteem-enhancing collective values (Merari et al., 2009). Disrespect for one’s social identity via marginalization or prejudice, as with some prevalent expressions of Islamophobia against Muslim citizens and immigrants in host countries, is one possible mechanism for such loss of personal significance and self-esteem (Kruglanski et al., 2014). From this vantage, our social exclusion manipulation may have activated participants’ motivation to enhance personal significance by increasing will to fight and die in defense of relevant in-group values and potentiating neural response to (what were initially) nonsacred values.

It is worth mentioning that our study concerned expressed willingness to fight in a sample vulnerable to recruitment into violent extremist groups and not actual costly sacrifices in active militants. However, given the direct relationship between sacred values and both expressed and actual willingness to fight and die in frontline combatants in our previous study (Gómez et al., 2017), the behavioral and brain relationships found in our current sample could well extend to active militants.

The sample size of 38 participants in the fMRI study was sufficient to detect neural effects that survived familywise error multiple comparison correction. As stated in the behavioral result section, the experimental manipulation led to large effect sizes across all standard manipulation checks. Thus, this study helps fill a gap in the radicalization literature. Recent meta-analyses show that what research on radicalization and violent extremism lacks are primary quantitative data, inferential statistics, and experimental design (Silke, 2008; Neumann and Kleinmann, 2013; Schuurman, 2018). This study contributes to all of those areas.

There are a number of limitations in the present study. Although the social exclusion manipulation has successfully evoked feelings of social exclusion in numerous laboratory settings (Zadro et al., 2004; Williams and Jarvis, 2006), it may be less effective in more complex real life situations. Thus, the fact that a 2-min online toss-ball game elicited neural effects associated with value sacredness, holds out the possibility that more powerful day-to-day social exclusion experiences could affect the brain in more dramatic ways. In addition, the present study does not allow for a direct comparison between our young vulnerable sample and individuals with well-established radicalized leanings. Thus, a comparison between groups matched for confounds such as nationality, degree of social integration, and so forth, or a longitudinal study, might be better suited for capturing a neural shift in sacred value processing throughout radicalization.

In sum, the combined evidence between the behavioral and neural responses to both sacred value processing and willingness to fight and die indicates that social exclusion in young and vulnerable individuals may increase similarities between nonsacred values and sacred values in terms of heightened left inferior frontal activity and greater expressed willingness to fight and die. The findings point to social exclusion as a possible contributing factor to radicalization, in line with analyses from political science and criminology (Pickering et al., 2008; Wright-Neville and Halafoff, 2010). If so, then counteracting social exclusion and sacralization of values should be considered in any intervention or policy aimed at preventing radicalization.

## Author Contributions

The project director was SA. The field studies were conceived and designed by NH and SA with the participation of JG, HS, and RD. Ethnographic and behavioral data were collected by NH and analyzed by NH, HS, and SA. The fMRI studies were directed by OV and designed by CP with the participation of OV, AT, SA, NH, JG, and RD. The fMRI studies were carried out by CP, and the data were analyzed by CP and OV. The manuscript was written by CP, NH, SA, and HS. All authors reviewed the final manuscript. CP and NH contributed equally to this manuscript. SA and OV are the corresponding authors.

### Conflict of Interest Statement

The authors declare that the research was conducted in the absence of any commercial or financial relationships that could be construed as a potential conflict of interest.
