# Supplementary material for: Neural and Behavioral Correlates of Sacred Values and Vulnerability to Violent Extremism
Source: Front Psychol. 2018 Dec 21;9:2462. doi: 10.3389/fpsyg.2018.02462 (PMC6309619; doi:10.3389/fpsyg.2018.02462)
Supplement: Supplementary file 1 [file Data_Sheet_1.docx]

**Supplementary material (Pretus, Hamid et al.)**

**Neuroimaging results**

As a parametric regressor, willingness to fight and die for both sacred and non-sacred values was negatively associated with activity in: the right superior medial and middle frontal gryus (T = 5.27, p < 0.001 FWEc, single voxel p < 0.001), right superior parietal (T = 5.32, p < 0.001 FWEc, single voxel p <0.001), and left putamen (T = 5.27, p = 0.047 FWEc, single voxel p < 0.001). Thus, the higher the willingness to fight and die score for a given value, the lower the activity in said areas (Figure S1 and Table S2).

**Table S1.** Results of the neural analysis including the within-subject sacred and non-sacred value conditions each modelled by familiariy, salience, attiude strength and emotional instensity scores as a parametric regressor and social exclusion vs. non-exclusion as a between-subjects factor. (*) small volume correction using left inferior frontal gryus mask extracted from the whole sample sacred vs. non-sacred value contrast of the main GLM.

| **N = 28** | **Region label (aal)** | **MNI coordinates** | | | **K** | **T max** | **p - value** |
| --- | --- | --- | --- | --- | --- | --- | --- |
|  |  | **x** | **y** | **z** |  |  |  |
| **Sacred > non-sacred values** | |  |  |  |  |  |  |
| **Whole sample** | L inferior frontal gryrus (pars triangularis ) | -46 | 26 | -2 | 87 | 3.95 | 0.005* |
| **Included > excluded** | L inferior frontal gryrus (pars triangularis ) | -38 | 42 | 12 | 6 | 4.28 | 0.026* |
| **Excluded > included** | - |  |  |  |  |  |  |

**Table S2.** Results of the neural analysis including the effect of willingness to fight and die for both sacred and non-sacred values as a parametric regressor.

| **N = 38** | **Region label (aal)** | **MNI coordinates** | | | **K** | **T max** | **p - value** |  |
| --- | --- | --- | --- | --- | --- | --- | --- | --- |
|  |  | **x** | **y** | **z** |  |  |  | |
| **Will to fight and die (negative correlation)** | | |  |  |  |  |  | |
| **Whole sample** | L calcarine | -12 | -82 | 4 | 5462 | 9.18 | < 0.001 | |
|  | L putamen/L insula/L caudate | -22 | 18 | -2 | 301 | 5.33 | 0.047 | |
|  | R superior parietal | 20 | -62 | 66 | 942 | 5.32 | < 0.001 | |
|  | R superior medial frontal gyrus/R superior and middle frontal gyrus | 12 | 32 | 44 | 3155 | 5.27 | < 0.001 | |
|  | R postcentral | 48 | -6 | 44 | 547 | 4.96 | 0.004 | |
|  | L Heschl/L insula | -30 | -30 | 8 | 514 | 4.72 | 0.005 | |


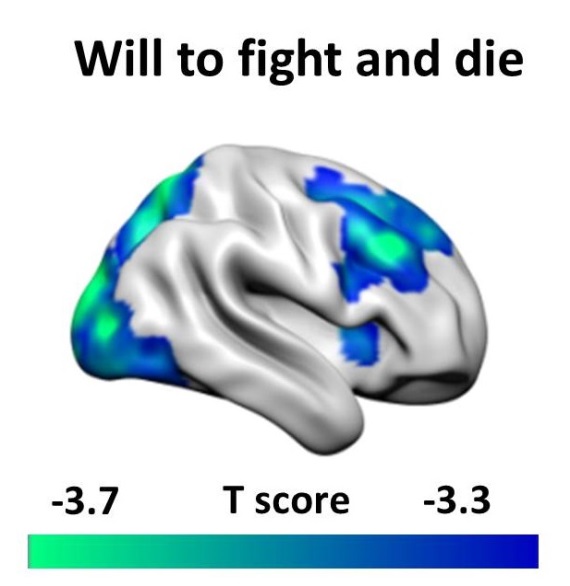


**Figure S1.** Neuroimaging results associated with willingness to fight and die including lower activity in the the right superior medial and middle frontal gryus (T = 5.27, p < 0.001 FWEc, single voxel p < 0.001), right superior parietal (T = 5.32, p < 0.001 FWEc, single voxel p <0.001) and left putamen (T = 5.27, p = 0.047 FWEc, single voxel p < 0.001).
